# Successful treatment of pleural empyema and necrotizing pneumonia caused by methicillin-resistant *Staphylococcus aureus* infection following influenza A virus infection: A case report and literature review

**DOI:** 10.3389/fped.2022.959419

**Published:** 2022-08-26

**Authors:** Chunjiao Han, Tongqiang Zhang, Yidi Zhao, Lili Dong, Xiaole Li, Jiafeng Zheng, Wei Guo, Yongsheng Xu, Chunquan Cai

**Affiliations:** ^1^Clinical School of Pediatrics, Tianjin Medical University, Tianjin, China; ^2^Department of Pulmonology, Tianjin Children’s Hospital, Tianjin University Children’s Hospital, Tianjin, China; ^3^Institute of Pediatrics, Tianjin Children’s Hospital, Tianjin University Children’s Hospital, Tianjin, China

**Keywords:** *Staphylococcus aureus*, influenza A virus, pleural empyema, necrotizing pneumonia, bronchoscopy

## Abstract

With the rapid increase in the number of infections, children with *Staphylococcus aureus* (*S. aureus*) infection secondary to Influenza A virus (IAV), appear to have a great possibility of causing severe complications and illness. Despite some cases and research findings regarding the death of children with IAV and *S. aureus*, coinfection included, there were few details about successful treatment of pleural empyema and necrotizing pneumonia caused by methicillin-resistant *Staphylococcus aureus* (MRSA) infection following IAV. In this case report, we describe the clinical symptoms and treatment of a teenager with pleural empyema and necrotizing pneumonia related to *S. aureus* secondary infection who was initially infected by IAV. This case highlights the importance of early recognition and application of thoracoscopy for this potentially fatal pleural empyema caused by MRSA and IAV coinfection. We conclude that this is a significant case that contributes to raising awareness regarding rarely occurring severe respiratory infections by MRSA in a child with normal immune function after IAV. In addition, further studies are needed to explore risk factors for IAV coinfection with *S. aureus*.

## Introduction

Retrospective studies of samples from the four pandemic influenza outbreaks of the last century have identified secondary bacterial infections as the fatal cause of co-morbidity and co-mortality, which reportedly manifested especially in the following week after viral infection symptoms are manifested, in 40–95% of influenza A -associated cases (1). IAV infections are associated with increased susceptibility to secondary bacterial infections, such as *S. aureus* and *Streptococcus* infections, wherein morbidity and mortality increase significantly (2). Children with IAV and *S. aureus* coinfection appear to have a great possibility of causing severe complications, and the mortality rate is very high as well. However, the details about the successful treatment of complications caused by coinfection of IAV and *S. aureus* have been rarely reported. In the present study, we report a unique case of pleural empyema and necrotizing pneumonia related to MRSA in a 13-year-old boy who was initially infected by IAV. Meanwhile, based on previous literature reports, we summarized the clinical characteristics and treatment of IAV and *S. aureus* coinfection.

## Case description

**FIGURE 1:**
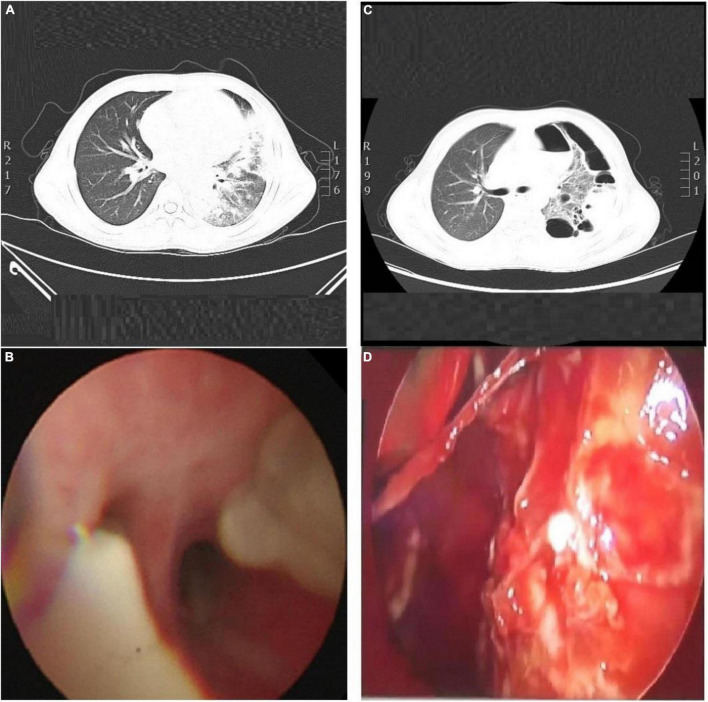
**(A)** CT scan of his chest, show the left lung and the right lower lobe consolidation, with atelectasis in the lower lobe of the left lung. The left pleural cavity showed a small amount of pleural effusion, and the lumen of the bronchial branch of the left lower lobe was not unobstructed. **(B)** Bronchoscopy, show the basal segment of left lower lobe, the bronchial mucosa was rough, a large number of yellow and white mucus plugs were found in the opening, and the ventilation was not smooth. **(C)** Chest CT scan. Indicate consolidation of left and right upper and lower lobes with signs of atelectasis and multiple cavities in the left lung, partially wrapped left pneumothorax. **(D)** Thoracoscopy, show the lung surface covered with a yellow purulent moss-like layer.

**FIGURE 2:**
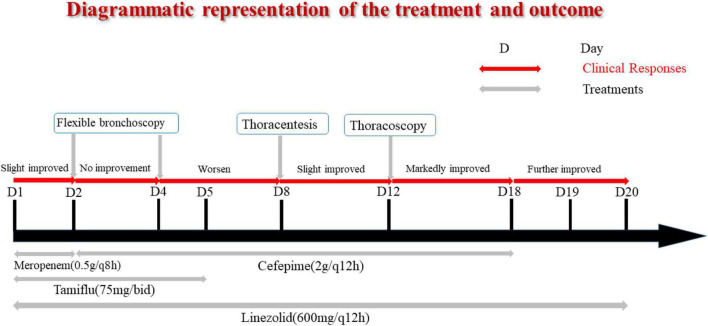
Diagrammatic representation of the treatment and outcome.

A previously healthy 13-year-old boy was hospitalized with a three-day history of cough and fever. He was diagnosed with IAV infection in the local hospital. On admission, he was in respiratory distress and complained of left-sided chest pain. Laboratory indicators were shown in Table 1. His oxygen saturation level was 89–94% with an oxygen supply and he could not even lie down. The computed tomography (CT) scan of his chest showed atelectasis and a small amount of pleural effusion (Figure 1A). He was provided with oxygen support, anti-infection therapy and nutritional support. A diagrammatic representation of the treatment and outcome was presented in Figure 2.

**TABLE 1:** Changes of various laboratory values.

Date of examination	WBC (×10^9^/L; 4.0–10.0)	Neutrophils (%; 45–77)	CRP (mg/L; 0–8)	Hb (g/L; 110–160)	PLT (×109/L; 100–300)	PCT (ng/ml)	IL-6 (pg/ml)	D-dimer (mg/L)	CK (U/L; 50–310)	CKMB (U/L; 0–24)	LDH (U/L; 120–300)	AST (U/L; 15–40)
1st	6.9	8.14	12.33	135	188	NA	NA	NA	NA	NA	NA	NA
4th	5.23	88	237.1	126	117	NA	NA	NA	508	20	355	33
5th	5.78	91.2	202.8	151	115	49.12	424.6	NA	Normal	NA	NA	NA
6th	6.65	82	64.4	124	163	8.66	NA	0.2	NA	NA	458	NA
8th	11.77	72	14.7	136	248	NA	NA	NA	NA	NA	NA	NA
11th	19.27	89	17.9	150	426	0.5	31.59	0.4	NA	NA	355	NA
13th	18.86	82	45	135	299	NA	NA	NA	NA	NA	NA	NA
16th	11.26	77	66.2	129	283	NA	NA	NA	NA	NA	NA	NA
18th	7.96	74	22.2	111	298	NA	NA	NA	NA	NA	NA	NA
19th	7.05	66	10.4	116	318	0.09	13.44	0.9	NA	NA	NA	NA
22th	4.69	60	<2.5	114	299	NA	NA	NA	NA	NA	NA	NA

WBC, white blood cell; PCT, procalcitonin; CRP, c-reactive protein; LDH, lactate dehydrogenase; AST, aspartate aminotransferase; CK, creatine Kinase; CKMB, creatine kinase Mb isoenzyme.

The following day, the patient was not doing well and developed a stridor; hence, the decision was made to perform a flexible bronchoscopy with bronchoalveolar lavage (BAL) under ECG monitoring and respiratory oxygen support after employing local anesthesia (Figure 1B). Because the patient’s severe pneumonia progressed rapidly and even endangered his life, an early and accurate etiological diagnosis was very important for the implementation of pathogen-specific treatment. Therefore, the next-generation sequencing analysis from BAL fluid was performed, and it indicated *S. aureus* as the infectious pathogen residing in the patient’s lungs. Blood culture for microbial infection was also found positive for *S. aureus*. On the fourth day of hospitalization, the chest X-ray showed no improvement, so the bronchoscope was continued to be used for BAL. Tracheal aspirate showed heavy growth of MRSA sensitive to linezolid, vancomycin, and rifampicin. Meanwhile, it was resistant to tetracyclines and quinolones.

Unfortunately, the patient was very ill, remained pyrexial (38.8°C) and developed a left-sided pyopneumothorax on day 8 of hospitalization with recurring fever, and the body temperature increased to 38.8°C. The patient complained of chest pain related to breathing abnormality, accompanied by apparent breath-holding. B-scan ultrasound and CT imaging (Figure 1C) of thorax revealed massive bilateral pleural effusions and multiple cavities in the left lung. Based on the laboratory and imaging diagnostic investigations, thoracentesis was performed on the patient’s left chest, and the chest tube was placed on the same side.

Even after 12 days of hospitalization and treatment, the patient’s condition did not improve due to poor drainage of empyema as a result of cellulose and thick pus accumulation. The patient underwent thoracoscopy to diagnose and treat pleural effusion (Figure 1D). After thoracoscopic investigations of the pleural cavity, the lung surface was cleared of the thick yellow moss-like layer of pus, and the left chest cavity was repeatedly flushed until the outflow was clear. At the end of the surgical procedure, the light bloody liquid could be seen in the chest bottle, and the chest tube was connected to the closed thoracic drainage system to prevent further pleural effusions.

On the 19th day of hospitalization, the chest drainage tube was removed. His cough condition was improved, and his breathing rate was stable without the occurrence of shortness of breath. The patient was discharged without any complaints of breathing discomfort. The patient was followed up for a chest X-ray examination after ten months of hospital discharge. The prognosis was good and the patient was in good physical condition.

## Discussion

Influenza is considered as a known potential risk factor for Staphylococcal diseases (3). There are strong and consistent pieces of evidence of epidemiologically and clinically important interactions between the influenza virus and secondary bacterial respiratory pathogens (4). A study in the United States (5) showed that *S. aureus* was the most common bacterial pathogen (44%) among the 36 children who died of bacterial co-infection reported during the 2004–2007 influenza season, while MRSA accounted for 60%. The complication of co-infection progresses rapidly, and children cannot get accurate and timely treatment, which is one of the reasons for its high mortality rate.

There are several hypothetical mechanisms of bacterial co-infection secondary to influenza virus infection. It is reported that the influenza virus can increase the adhesion of bacteria by destroying the epithelial layer of the tracheobronchial tree and neuraminidase activity (6, 7). At the same time, Panton-Valentine leukocidin (PVL) is a pore-forming cytotoxin produced by *S. aureus* genes, acting synergistically to induce a strong lytic effect on host defense cells, notably with poly-morphonuclear leucocytes but especially on neutrophils (8). Previously infected influenza virus can enhance the proinflammatory and cytotoxic effects of PVL on neutrophils. Disintegration of the epithelial airway results in hemorrhage and tissue damage, which leads to the development of necrotizing pneumonia (7). In addition, nasal carriage of *S. aureus* is a significant risk factor for secondary staphylococcal pneumonia in IAV (9). Therefore, children with a history of *S. aureus* infection should be more alert to the risk of IAV and *S. aureus* co-infection. Finelli et al. found that compared with children without *S. aureus* infection, children with *S. aureus* infection were more prone to pneumonia and acute respiratory distress syndrome during the influenza season (5).

We reviewed 16 clinical reports of IAV and *S. aureus* coinfection; however, five of them lack detailed clinical information (Table 2). Reviewing the literature of 16 children with IAV and *S. aureus* co-infection, we can find that *S. aureus* is almost always characterized by MRSA. Most of these children are older than 10 years old, which is consistent with the result of Finelli et al. (5). Complications in these children mainly include sepsis, empyema, DIC, pneumothorax, lung abscess, emphysema, necrotizing pneumonia, and so on. However, most detection of virulence factors of *S. aureus* were missing. Two cases presented PVL (+) and one presented PVL (−). A study suggested that an early confirmed presence of the PVL toxin is particularly important in choosing antibiotics and administrating immunoglobulin that inhibits PVL toxin release in early stage (10). Notably, the application of bronchoscopy was important for early detection of the patient’s respiratory conditions. However, bronchoscopic results have rarely been reported and utilized in treating children with *S. aureus* co-infection that is secondary to IAV. In this case, we removed the obstruction of the trachea and lung with the assistance of bronchoscopy findings. Moreover, we could wash the diseased area of alveoli through alveolar lavage and subsequently analyzed the BAL fluid for bacterial infection. Bronchoscopy has also been reported in the case of Sharp et al. (12). Copious cloudy secretions, fibrinous debris, and patchy plaques were found in the main bronchi and distal trachea. We should pay more attention to the application opportunity of bronchoscope. Thoracoscopy was also the key for this potentially fatal pleural empyema caused by MRSA and IAV coinfection when patient developed pleural adhesion and multiple cavities. In addition, the application of drugs was also critical. In our case, we covered the positive cocci in the initial treatment. We timely adjusted antibiotics by detecting the drug resistance of *S. aureus*, which effectively controlled the *S. aureus* infection. Due to the application of flexible bronchoscopy, thoracoscopy and adequate anti-infective treatment, we could successfully relieve the patient’s uncomfortable respiratory conditions, and let the patient be discharged within 3-weeks of hospitalization.

**TABLE 2:** The clinical characteristics and results of the literature review of the IAV and *S. aureus* coinfection.

References	Cases in article	Age at the diagnosis	Sex	Duration of hospitalization (days)	Complication		Drug	Outcome	*S. aureus* drug resistance	Virulence factor
Boettger et al.(11)	1	21-month-old	NA	3	Sepsis, atelectasis, and pleural effusion	Orotracheal tube, central venous catheter insertion, and ventilatory support	Oseltamivir, Ceftriaxone, and clarithromycin	Death	MRSA	icaA, icaB, icaD, SeiO, hla and hlb(+); PVL and TSST-1(−)
Sharp et al.(12)	1	9-year-old	Male	12	Sepsis and circulatory collapse	ECMO, bronchoscopy	Vancomycin, meropenem, and enteral oseltamivir	Death	MRSA	NA
Pugh(13)	1	5-year-old	Male	30 more	Empyema	Chest tube insertion	Vancomycin, clindamycin, and cefepime	Recovery	MRSA	NA
Thomas et al. (14)	1	13-year-old	Female	8	Acute necrotizing tracheobronchitis	None	Amoxicillin and codeine	Death	Penicillin susceptible	NA
Obando et al.(15)	1	12-year-old	Male	28	Empyema and pneumothorax	Thorascopic surgery and chest tube insertion	Ceftriaxone, vancomycin, clarithromycin, linezolid, and oseltamivir	Recovery	MRSA	PVL(+), ACME(+)
Barrett et al.(16)	1	17-year-old	Male	17	Sepsis	None	Clarithromycin and co-amoxiclav	Recovery	MSSA	PVL(+)
Thomas et al.(17)	1	8-month-old	Male	25	Pleural effusion	Chest tube insertion	Ampicillin sodium, gentamicin sulfate, and nafcillin sodium	Recovery	NA	NA
Tanaka et al.(18)	1	11-month-old	Female	37	Empyema, DIC	Thoracentesis and thoracoscopic decortication	Oseltamivir phosphate	Recovery	MSSA	NA
CDC (19)	3	10-year-old	Male	3	Hypotension and hypoxia	Endotracheal intubation	Ceftriaxone and vancomycin	Death	MRSA	NA
		14-year-old	Male	2	Sepsis, DIC, and necrotizing pneumonia	Endotracheal intubation	Clarithromycin, penicillin, oseltamivir, ceftriaxone, and vancomycin	Death	MRSA	NA
		8-year-old	Female	20	Renal and hepatic failure, a subpulmonic abscess, respiratory distress, and sepsis	En dotracheal intubation	Azithromycin, dexamethasone, and ceftriaxone	Death	MRSA	NA
CDC(20)	5	14-year-old	Female	19	NA	NA	Oseltamivir	Death	MRSA	NA
		13-year-old	Male	5	NA	NA	Oseltamivir	Death	MRSA	NA
		15-year-old	Male	2	NA	NA	None	Death	NA	NA
		9-year-old	Female	15	NA	NA	Oseltamivir	Death	MRSA	NA
		15-year-old	Male	7	NA	NA	Oseltamivir	Death	MRSA	NA

In conclusion, due to the synergistic pathogenic effects between the influenza virus and coinfecting respiratory bacteria, we should raise awareness regarding the rarely occurring severe respiratory infections by *S. aureus*, following influenza for early diagnosis and rapid recovery from respiratory complications in children. In addition, this study suggests a need for further research about risk factors of secondary *S. aureus* infection of IAV.

## Data availability statement

The original contributions presented in this study are included in the article/supplementary material, further inquiries can be directed to the corresponding author/s.

## Ethics statement

Written informed consents were obtained from the parents for publication of this report.

## Author contributions

TZ and XL cared for patients. YZ and LD collected the data. CH and JZ drafted the article. WG and YX revised it for intellectual content. CH and CC approved the final completed article. All authors read and approved the final manuscript.
